# Exploring the role of CD8 ^+^ T cells in clear renal cell carcinoma metastasis

**DOI:** 10.1002/2211-5463.13819

**Published:** 2024-06-13

**Authors:** Yuanhong Chen, Jiajia Shen, Caixia Ling, Zhengfang Liang, Shaoang Huang, Wenxian Lin, Yujuan Qin, Lingzhang Meng, Yanhong Luo

**Affiliations:** ^1^ Center for Systemic Inflammation Research (CSIR), School of Preclinical Medicine Youjiang Medical University for Nationalities Baise China; ^2^ Department of Pathogenic Biology and Immunology Youjiang Medical University for Nationalities Baise China; ^3^ Modern Industrial College of Biomedicine and Great Health Youjiang Medical University for Nationalities Baise China; ^4^ Department of Urinary Surgery The Affiliated Hospital of Youjiang Medical University for Nationalities Baise China; ^5^ Department of Interventional Oncology Affiliated Hospital of Youjiang Medical College for Nationalities Baise China; ^6^ Institute of Cardiovascular Sciences Guangxi Academy of Medical Sciences Nanning China

**Keywords:** CD8^+^ T cells, immune cells infiltrated tumor, immunofluorescent, metastasis, renal cell carcinoma, transcriptome

## Abstract

Clear cell renal cell carcinoma (ccRCC) accounts for approximately 75–80% of all patients with renal cell carcinoma. Despite its prevalence, little is known regarding the key components involved in ccRCC metastasis. In this study, scRNA‐seq analysis was employed to classify CD8^+^ T cells into four sub‐clusters based on their genetic profiles and immunofluorescence experiments were used to validate two key clusters. Through gene set enrichment analysis, these newly identified sub‐clusters were found to exhibit distinct biological characteristics. Notably, TYMP, TOP2A, CHI3L2, CDKN3, CENPM, and RZH2 were highly expressed in these sub‐clusters, indicating a correlation with poor prognosis. Among these sub‐clusters, CD8^+^ T cells (MT‐ND4) were identified as potentially playing a critical role in mediating ccRCC metastasis. These results contribute to our understanding of CD8^+^ T cell heterogeneity in ccRCC and shed light on the mechanisms underlying the loss of immune response against cancer.

## Graphical abstract

In this study, we investigated CD8^+^ T cell heterogeneity in clear cell renal cell carcinoma (ccRCC) and identified key sub‐clusters and genes linked to metastasis. Notably, CD8^+^ T cells (MT‐ND4) were suggested to potentially drive ccRCC metastasis. These findings contribute to our understanding of CD8^+^ T cell diversity in ccRCC and the mechanisms behind the compromised immune response to cancer.

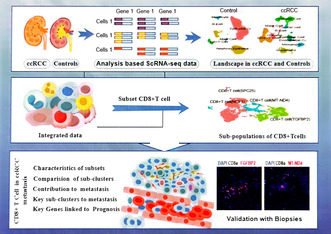

**Abbreviations**
avg log2FC: each gene's average fold change
ccRCC: clear cell renal cell carcinoma
DEGs: differentially expressed genes
GSEA: gene set enrichment analysis
TME: tumor immune microenvironment

Renal cell carcinoma (RCC) is a prevalent urologic malignancy that occurs only as a result of bladder and prostate cancer. Statistics show that clear cell renal cell carcinoma (ccRCC) accounts for approximately 75–80% of all patients with RCC [1, 2], with a death rate of roughly 85% of all diagnosed cases. The tumor immune microenvironment (TME) is a complex ecosystem that plays a crucial role in cancer initiation and progression, as well as functioning as an immunotherapy interface [3]. Large‐scale genomic investigations on ccRCC have revealed critical insights into TME changes that influence tumor growth and response to immune checkpoint inhibition [4, 5]. Tumor cells and nontumor components such as neutrophils, B cells, fibroblasts, cytokines, endothelial cells, monocytes, macrophages, and CD8^+^ T cells are all part of the TME [6]. Neutrophils are resistant to ipilimumab and pembrolizumab anticancer immune treatments due to low/no expression of PD‐1, PD‐L1, and CTLA‐4 [7]. Endothelial cells promote metastasis by increasing the expression of S100A4, THY1, and ACTA2 [8]. Fibroblasts remodeled the extracellular matrix in the TME towards invasion and metastasis by expressing POSTN [9]. Even though CD8^+^ T cells have long been regarded to be a critical type of cell in the development and progression of malignancies, recent new immunotherapies targeting immunological checkpoints as the standard of care have altered the treatment paradigm of ccRCC [10, 11], a sizable CD8^+^ T cell fraction of patients with ccRCC do not respond to these treatments and those who do at first progress. Both healthy and diseased circumstances exhibit heterogeneous CD8^+^ T cells [12, 13, 14, 15]. Patients undergoing immunotherapy for ccRCC still face significant obstacles due to the heterogeneity of CD8^+^ T cells, which roles in ccRCC immunization responses, metastasis, and prognosis have yet to be fully elucidated. In this study, CD8^+^ T cells were subdivided into four sub‐clusters, NCF1, FGFBP2, MT‐ND4, and SPC25, all of which failed to destroy tumor cells, resulting in metastasis. Our research elucidated the heterogeneity of CD8^+^ T cell sub‐populations and identified crucial sub‐clusters and genes associated with metastasis.

## Materials and methods

### Bioinformatics analysis based on scRAN‐seq

The scRAN‐seq data for ccRCC and control biopsy tissues were downloaded from the NCBI GEO public database (https://www.ncbi.nlm.nih.gov/geo/) with the following login codes GSE131685 [16] and GSE171306 [17]. In total, three ccRCC tissues and three normal tissues were obtained. The data were normalized using the R package SCTransform and then clustered at a resolution of 0.6 and plotted using the r package seurat (v4.0.4, New York Genome Center, New York City, NY, USA) for UMAP. Cells with differentially expressed genes (DEGs) were processed for analytical assessment with the r package enhancedvolcano (v1.10.0). Gene Set enrichment analysis (GSEA) was computationally analyzed with the r package cluster profiler (v4.0.0).

### Human biopsies

Biopsies were extracted from individuals with ccRCC who had surgery to validate the CD8^+^ T cell sub‐populations. Following the pathological inspection, the leftover samples were processed for immunofluorescent staining. This study obtained written informed consent from all participants before commencement. The research protocol underwent ethical review and approval by the Youjiang Medical University Ethics Committee for Nationalities (#2023011601), ensuring adherence to their guidelines and regulations. Additionally, all experiments strictly followed the principles outlined in the Declaration of Helsinki.

### Immunofluorescent microscopy

Biopsies were embedded in a 10 × 10 × 5 mm mold (Sakura, #4565, Tokyo, Japan) and sectioned to a thickness of 6 μm with a microtome (LEICA CM1950, Wetzlar, Germany). After fixation in methanol at −20 °C, the sections were washed twice in PBS. The sections were then blocked in PBS/0.5%BSA/Fcγ receptor blocker for 1 h at 4 °C. The slices were incubated with primary antibody at 4 °C overnight and washed 3 times with PBS, incubated with fluorescently labeled secondary antibody, and washed 2 more times with PBS, finally, the sections were mounted with Fluoromount‐G (SouthernBiotech, #0100‐01, Birmingham, AL, USA).

The primary antibodies employed for immunofluorescent examination were: CD3e Mouse anti‐human/mouse (IgG2b) (Invitrogen, #MA5‐31463, Carlsbad, CA, USA), Rabbit‐anti‐human FGFBP2 (IgG) (Invitrogen, #PA5‐59687), Rabbit‐anti‐human/mouse/rat MT‐ND4 (Invitrogen, #PA5‐116791), A488 Donkey‐anti‐mouse IgG(H + L) (Invitrogen, #A21202) and Cy3 Goat‐anti‐rabbit IgG(H + L) (Affinity Bioscience, #S0011, Nanjing, Jiangsu, China) were employed as secondary antibodies in this study.

## Results

### Identification of CD8 ^+^ T cells

**Fig. 1:**
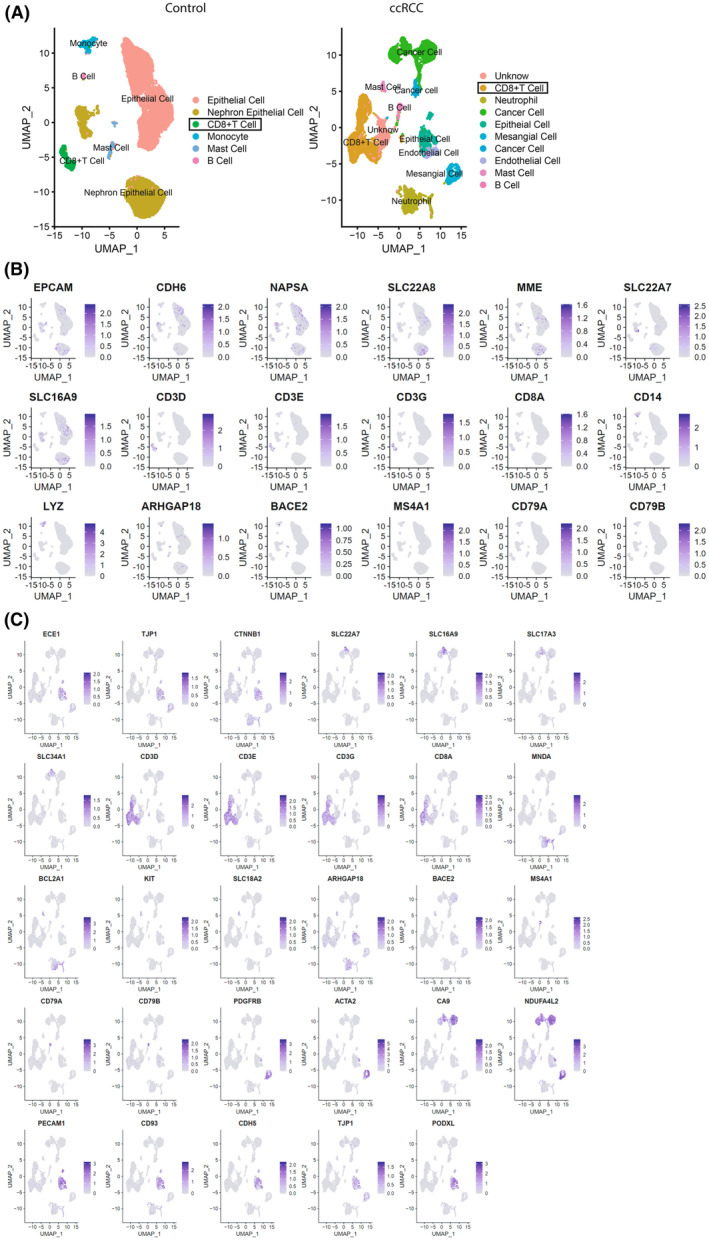
Identification of CD8^+^ T cells in ccRCC biopsies and scRNA‐seq analysis showed four sub‐clusters in kidney‐associated biopsies. (A) UMAP plots revealed six cell types in normal human kidneys and 10 cell types in ccRCC biopsy tissues. The highlighted cell types represent CD8^+^ T cells. (B) In the control group, the Featureplot displayed the expression pattern of CD8^+^ T cell markers, including CD3D, CD3E, CD3G, and CD8A. Epithelial cells were annotated based on their canonical features EPCAM, CDH6, NAPSA, SLC22A8, and MME. Nephron epithelial cells were identified by the expression pattern of “SLC22A7” and “SLC16A9.” Master cells were classified by their expression of “ARHGAP18” and “BACE2.” Monocyte cells were characterized by the marker genes “CD14” and “LYZ,” while B cells were identified by the marker genes “MS4A1,” “CD79A,” and “CD79B.” (C) In the ccRCC group, the feature plot displayed the expression pattern of CD8^+^ T cell markers, including CD3D, CD3E, CD3G, and CD8A. Cancer cells were identified based on their significant expression of CA9, CTR2, and NDUFA4L2. Epithelial cells were characterized by marker genes such as “ECE1,” “TJP1,” “CTNNB1,” and “PODX1.” Nephron epithelial cells were marked by genes like “SLC22A7,” “SLC16A9,” “SLC17A3,” and “SLC34A1.” Neutrophils had marker genes “MNDA” and “BCL2A1.” Master cell marker genes included “KIT,” “SLC18A2,” “ARHGAP18,” and “BACE2.” B cells were characterized by genes “MS4A1,” “CD79A,” and “CD79B.” Mesangial cells had marker genes “PDGFRB” and “ACTA2.” Endothelial cells were marked by genes such as “PECAM1,” “CD93,” “CDH5,” “TNNB1,” “TJP1,” and “PODXL.”

**Fig. 2:**
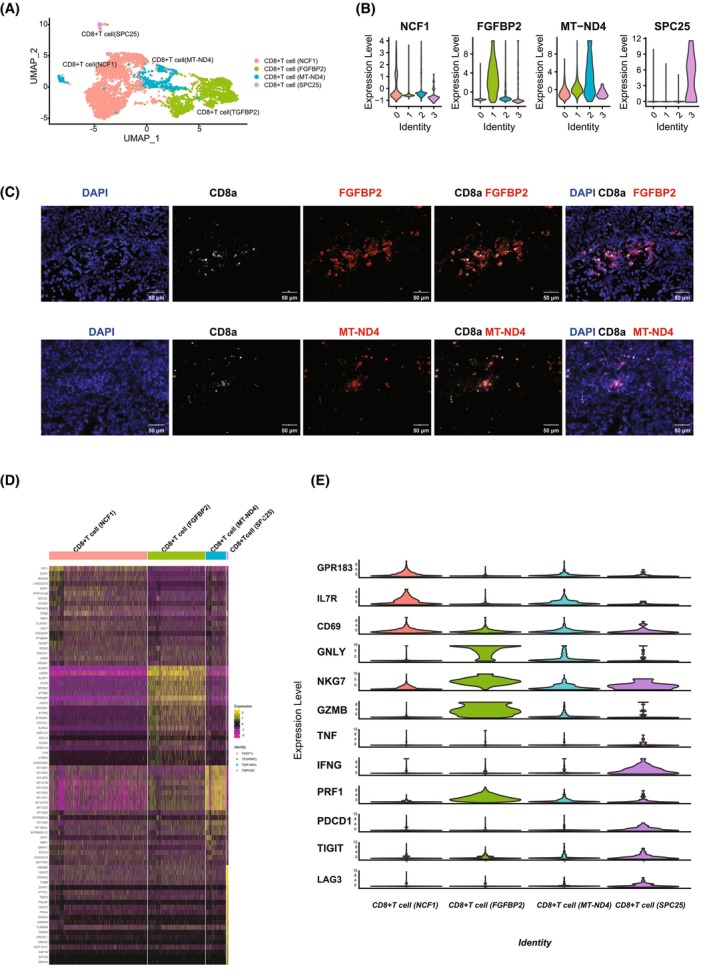
scRNA‐seq analysis showed four sub‐clusters in kidney‐associated biopsies. (A) UMAP map demonstrating the integration of CD8^+^ T lymphocytes derived from normal kidney and ccRCC tissues. Based on genetic characteristics, four sub‐clusters were discovered. (B) Violin plots depicted the expression patterns of four feature genes: NCF1, FGFBP2, TMT‐ND4, and SPC25. (C) Immunofluorescent labeling of two of these CD8^+^ T cell subsets: CD8^+^ T cell (FGFBP2), and CD8^+^ T cell (MT‐ND4). CD3e was used to mark T cells. (D) Heatmap showing the top 20 genes from each CD8^+^ T cell sub‐expression cluster's pattern. (E) Violin plots of notable individual genes showing the expression level distribution revealed that each CD8^+^ T cell sub‐cluster possesses distinct biological features.

With the advantages of the scRNA‐seq technique, cells from healthy kidneys were divided into 6 clusters; while in ccRCC biopsies, 10 cell clusters were identified [16, 17]. (Fig. 1A). In the control group, epithelial cells were annotated based on their canonical features EPCAM, CDH6, NAPSA, SLC22A8, MME (Fig. 1B), nephron epithelial cells were identified by the expression pattern of “SLC22A7,” “SLC16A9” (Fig. 1B), master cells were classified by their expression of “ARHGAP18,” “BACE2” (Fig. 1B). In ccRCC group, cancer cells were annotated by their significant expression of CA9, CTR2, NDUFA4L2 (Fig. 1C). In both groups, CD8^+^ T cells were identified by the expression of CD3D, CD3E, CD3G, and CD8A [18]. To analyze and compare CD8^+^ T cells between these two groups, integration was performed after CD^+^ T cells isolation from the above two groups. The sub‐cluster numbers of integrated CD8^+^ T cells varied depending on the “resolution” used in the Seurat package. To find the optimal sub‐cluster numbers, the r package multik was adopted [19], and found either 4 or 15 sub‐clusters should be the optional choice. Since the total CD8^+^ T cell numbers are not sufficient for 15 sub‐clusters, we choose to subdivided them into 4 clusters (Fig. 2A). During sub‐clustering, 172 genes were found preferentially expressed in the first sub‐cluster, 194 genes in the second sub‐cluster, 162 genes in the third sub‐cluster, 208 genes in the fourth sub‐cluster (Table S1). These sub‐clusters were also characterized by the preferential expression of NCF1, FGFBP2, MT‐ND4, and SPC25, respectively (Fig. 2B). The existence of these four sub‐clusters was further validated through immunofluorescence staining of the ccRCC biopsies (Fig. 2C). To better understand the biological/pathological role of these 4 newly identified CD8^+^ T cells, differentially expressed genes (DEGs) were calculated (Table S1) and the heatmap briefly showed the similarities/disparity of the top 20 genes of these 4 sub‐clusters (Fig. 2D). With the DEGs identified, comparative biological theme analysis was performed (Table S2). All four clusters played important roles in the progression of glomerulonephritis and kidney neoplasm. Specifically, CD8^+^ T cells (NCF1) were found to be potentially more crucial in the progression of renal angiomyolipoma and unilateral renal agenesis. CD8^+^ T cells (FGFBP2) demonstrated a potentially greater significance in mediating renal ischemia. Lastly, CD8^+^ T cells (MTND4) were identified as playing a more significant role in acute renal insufficiency. Unfortunately, none of these 4 clusters expressed the co‐stimulatory signal CD28 and proliferation‐activating cytokine IL2 (Table S1) [20], and the expression of PDCD1 and CTLA‐4 which is critical for assisting immune therapy was inhibited. Interestingly, CD8^+^ T cell (NCF1) and CD8^+^ T cell (MT‐ND4) were discovered to express markers similar to effector memory T cell (TEM, IL7R, GPR183) [21]; both of them expressed the activation marker CD69, but expressed low levels of Cytotoxic effectors such as GNLY, GMZB, IFNG, TNF or PRF1 [11, 22]; CD8^+^ T cell (FGFBP2) was identified as a cytotoxic CD8^+^ T cells featured by the expression of NKG7, GNLY, GZMB, and PRF1 [21]; CD8^+^ T cell (SPC25) was genetically characterized as a dysfunctional or exhausted phenotype of the CD8^+^ T cell population (TIGIT, PDCD1, LAG3, FASLG) [23, 24] (Fig. 2E). KEGG analysis indicated that CD8^+^ T cell (MT‐ND4) potentially played an important role in the development and metastasis of ccRCC, nephrotic syndrome, glomerular disease, renal ischemia, ureteral stenosis, and renal metastases in ccRCC patients [25, 26] (Table S2).

### CD8 ^+^ T cell sub‐populations could potentially contribute to distant metastasis

**Fig. 3:**
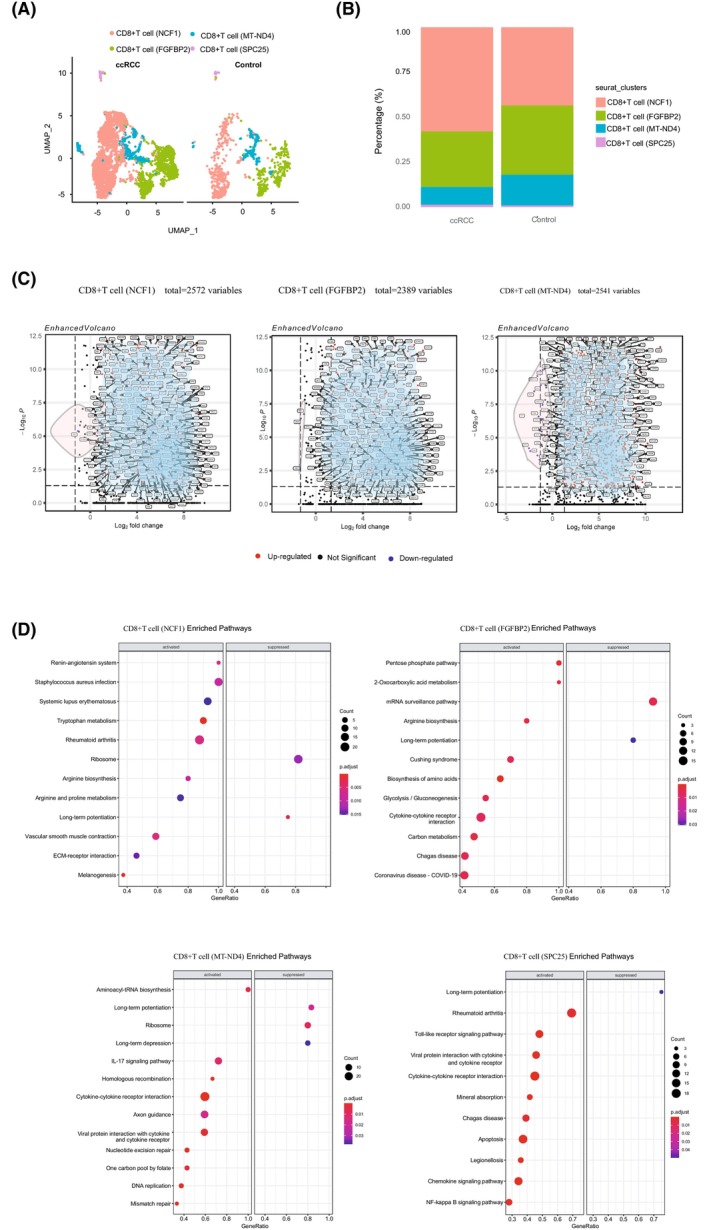
Comparison of CD8^+^ T cell sub‐clusters in ccRCC and normal human kidney tissue. (A) Split UMAP map demonstrated the location of CD8^+^ T cell sub‐clusters in normal and kidney samples. (B) A stacked bar plot was used to compare the frequency of CD8^+^ T cell sub‐clusters in normal and kidney biopsies. (C) Volcano plots depicted the distribution of DEGs derived from CD8^+^ T cell sub‐clusters CD8^+^ T cell (NCF1), CD8^+^ T cell (FGFBP2), and CD8^+^ T cell (MT‐ND4). (D) Dot plots revealed pathways that were up‐or down‐regulated in the CD8^+^ T cell sub‐clusters CD8^+^ T cell (NCF1), CD8^+^ T cell (FGFBP2), CD8^+^ T cell (MT‐ND4), and CD8^+^ T cell (SPC25).

Integrated scRNA‐seq data showed that the distribution and frequency of these sub‐populations were distinct from those of normal human renal CD8^+^ T cell sub‐populations (Fig. 3A,B). The CD8^+^ T cell immune response to ccRCC was inefficient or diminished, as evidenced by a significant decrease in the proportion of sub‐population CD8^+^ T cells (FGFBP2) and an increase in CD8^+^ T cells (NCF1). (Fig. 3B). This was resulted from an increase in effector memory CD8^+^ T cells and a decrease in cytotoxic CD8^+^ T cells. The results of further calculations showed that there were 2572 DEGs in CD8^+^ T cells (NCF1), 2389 DEGs in CD8^+^ T cells (FGFBP2) (ccRCC vs. normal human kidney), and 2541 DEGs in CD8^+^ T cells (MT‐ND4) (Fig. 3C). The DEGs from CD8^+^ T cell (SPC25) could not be determined due to extremely low numbers. GSEA analysis showed most of the pathways, including IL‐17 signaling (including IL17A, STAT3, CXCL2, and CXCL5) and cytokine‐cytokine receptor interaction signaling (including TGFBR3, IL17F, and IL10), were upregulated in ccRCC CD8^+^ T cell sub‐populations compared to normal human kidney tissues (Fig. 3D). All of these pathways are strongly associated with the development of cancer [27, 28, 29].

**Fig. 4:**
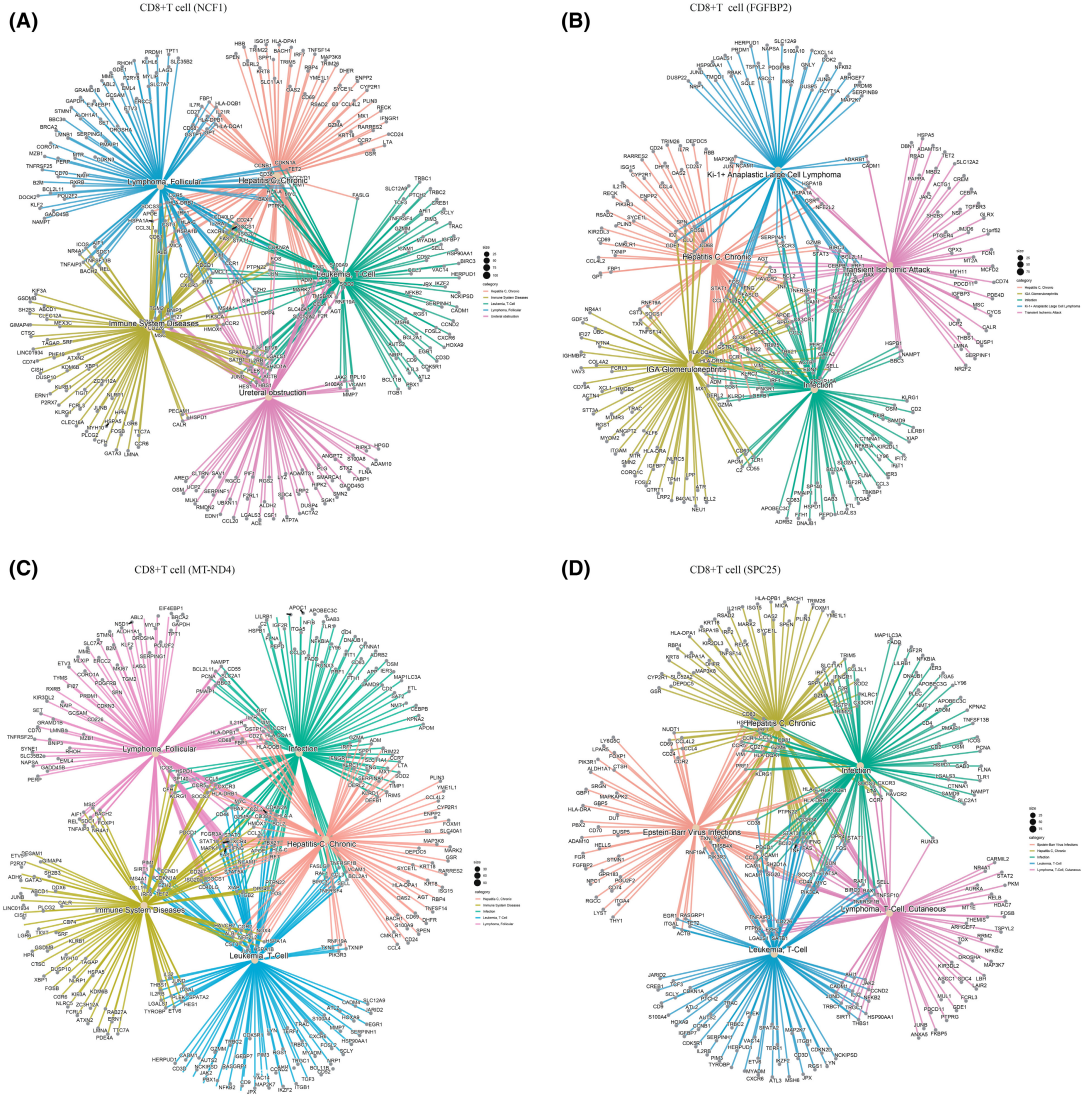
Contribution of CD8^+^ T cells in ccRCC biopsies to metastasis. (A) CNET plot depicts the CD8^+^ T cell (NCF1) DEGS and predicts metastases. (B) CNET plot depicts the CD8^+^ T cell (FGFBP2) DEGS and predicts metastasis. (C) CNET plot depicts the CD8^+^ T cell (MT‐ND4) DEGS and predicts metastasis. (D) CNET plot depicts the CD8^+^ T cell (SPC25) DEGS and predicts metastases.

**Fig. 5:**
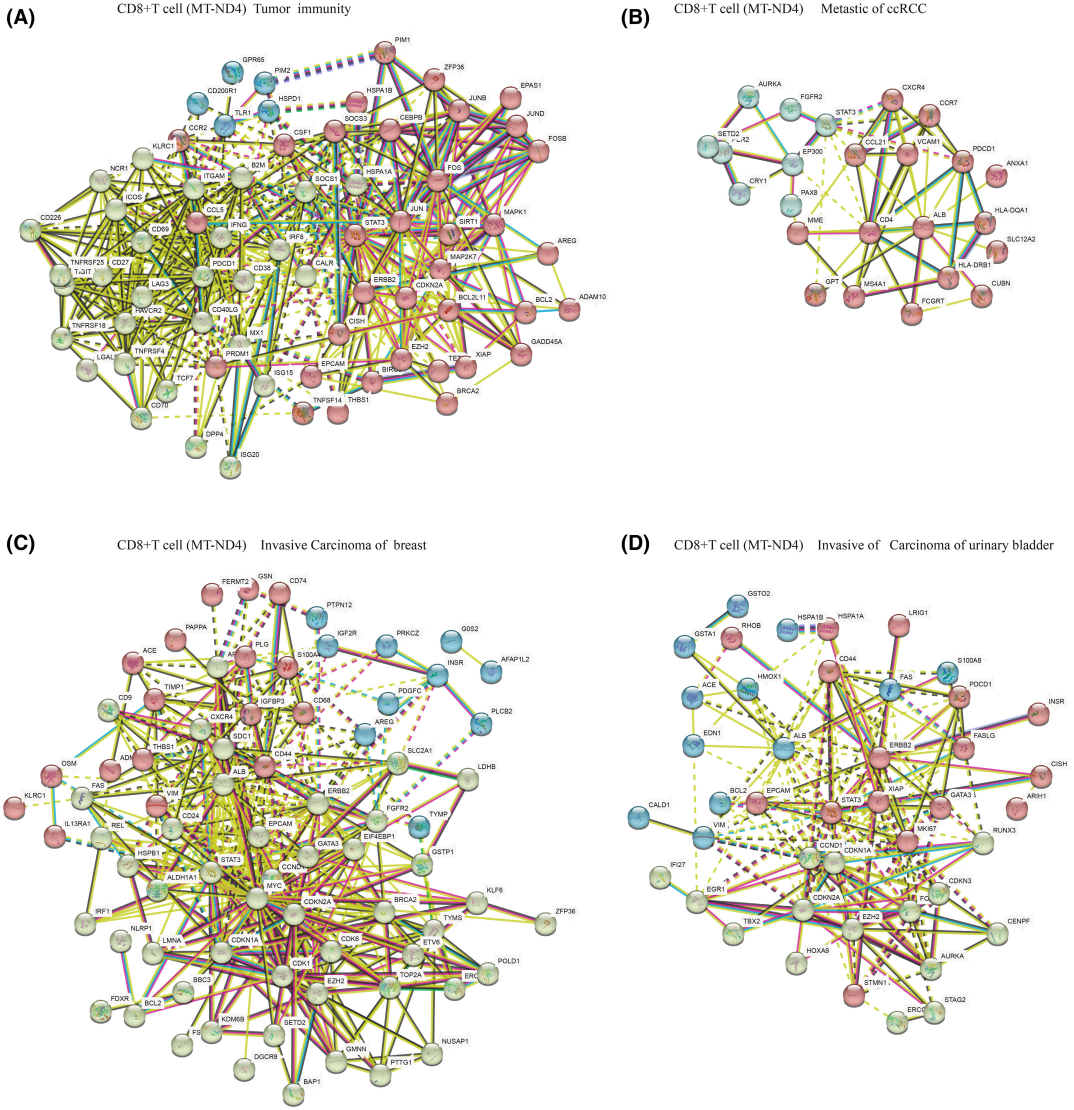
CD8^+^ T cell (MT‐ND4) in ccRCC biopsies contribute to metastasis. (A) Demonstration of tumor immune‐related protein interactions of ccRCC in CD8^+^ T cells (MT‐ND4) using the STRING tool. (B) Demonstration of metastasis‐related protein interactions of ccRCC in CD8^+^ T cells (MT‐ND4) using the STRING tool. (C) Demonstration of metastatic breast carcinoma‐related protein interactions of ccRCC in CD8^+^ T cells (MT‐ND4) using the STRING. (D) Demonstration of metastatic urinary bladder carcinoma‐related protein interactions of ccRCC in CD8^+^ T cells (MT‐ND4) using the STRING tool.

**Fig. 6:**
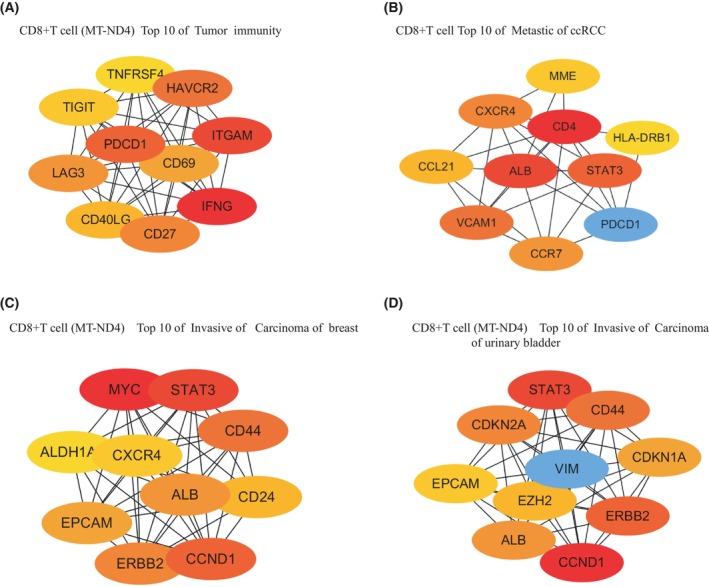
Top 10 metastasis‐related genes in CD8^+^ T cell (MT‐ND4). (A) Demonstration of top 10 tumor immune‐related genes in CD8^+^ T cells (MT‐ND4) using the cytoscape tool (Cytoscape Consortium, Seattle, WA, USA). (B) Demonstration of top 10 metastatic‐related genes of ccRCC in CD8^+^ T cell (MT‐ND4) using the cytoscape tool. (C) Demonstration of top 10 metastatic breast cancer‐related genes in CD8^+^ T cells (MT‐ND4) using the cytoscape tool. (D) Demonstration of top 10 metastatic urinary bladder cancer‐related genes in CD8^+^ T cells (MT‐ND4) using the cytoscape tool.

DEGs derived from the 4 CD8^+^ T cell sub‐populations observed above in ccRCC were subjected to a gene concept network analysis, CD8^+^ T cell (NCF1), CD8^+^ T cell (MT‐ND4), and CD8^+^ T cell (SPC25) were found to potentially contribute to ccRCC metastasis to Leukemia, Lymphoma, while CD8^+^ T cell (FGFBP2) appears to potentially contribute to ccRCC metastasis to Ki‐1^+^ Anaplastic Large Cell Lymphoma (Fig. 4A–D; Table S2). Among them, CD8^+^ T cells (MT‐ND4) were considered to be of significant importance in ccRCC patients. CD8^+^ T cells (MT‐ND4) were implicated in glomerular disease, tumor immunity, and tumor metastasis in ccRCC, including breast and bladder cancer, fibrosarcoma of the bladder, adenocarcinoma of the prostate, lymphoma, adult fibrosarcoma, and metastases from various other tumors (Fig. 4C, Table S2). To study the role of CD8^+^ T (MT‐ND4) cells in tumor immunity and metastasis, we employed STRING to analyze protein interactions and Cytoscape to display the top 10 core genes (Fig. 5, Fig. 6). Among these genes, PDCDC1 (Fig. 6A,B) is especially important in inhibiting cellular immunity, which is crucial for immune surveillance against cancer cells [22, 24]. In addition, STAT3 and MYC (Fig. 6B–D) have been shown to play significant roles in ccRCC cell metastasis through their regulation of cell proliferation, migration, invasion, and apoptosis [30].

### CD8 ^+^ T cells related genes contribute to distant metastasis

**Fig. 7:**
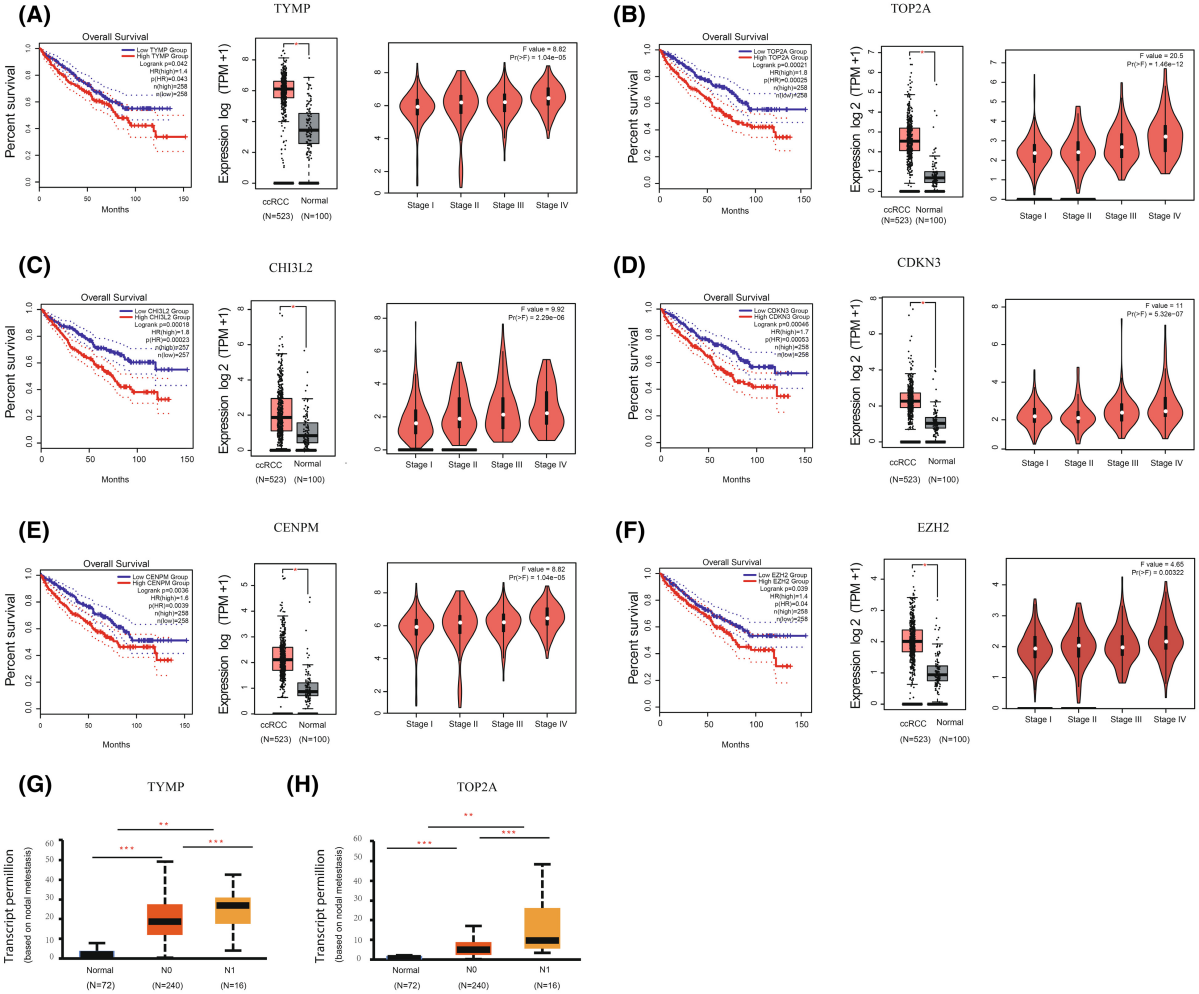
Identification of key genes linked to a poor prognosis. Following Cox regression analysis and Kaplan–Meier curves, survival curves unveiled six upregulated genes linked to poor prognosis in ccRCC patients, High Expression Group: *n* (high) = 258, Low Expression Group: *n* (low) = 258, with a 95% Confidence Interval. In each panel, the expression levels of the relevant genes were validated with the TCGA database, ANOVA was applied to compare matched TCGA normal samples with GTEx data, represented by box plots and stage plots, ccRCC: *N* = 523, Normal: *N* = 100, Mean ± SD, *P < 0.05, **P < 0.01, ***P < 0.001. Six genes were as follows (A) TYMP, (B) TOP2A, (C) CHI3L2, (D) CDKN3, (E) CENPM, (F) EZH2. (G) TYMP Gene Associated with Metastasis Based on nodal metastasis in ccRCC patients via the UALCAN was tested using *t*‐test. (H) TOP2A Genes Associated with Metastasis Based on nodal metastasis in ccRCC patients via the UALCAN was tested using *t*‐test.

All of the genes related to metastasis (Table S2) were tested in Gene Expression Profiling Interactive Analysis (GEPIA2) which was based on long‐term follow‐up studies of cancers. Among these, TYMP, TOP2A, CHI3L2, CDKN3, CENPM, and RZH2 were considered to be related to the unfavorable prognosis of ccRCC patients (Fig. 7). 2 of these genes (TYMP and TOP2A) were shown to be linked to distant metastases in ccRCC patients in terms of lymph node metastasis (Fig. 7).

### Statistical analysis

High‐throughput RNA‐seq data were analyzed using the deseq2 package, and visualizations were generated with ggplot2. Statistical analyses were performed using spss 24.0 software (IBM Corporation, Armonk, NY, USA). The Wilcoxon test was employed to compare differentially expressed genes (DEGs) within CD8^+^ T cell sub‐populations between ccRCC and control biopsy tissues. ANOVA was utilized to compare the expression levels of key genes through box plots and stage plots. Genes associated with nodal metastasis were identified using statistical analysis with the Student *t*‐test. Survival analysis was conducted with the survival package for Cox regression and Kaplan–Meier curves. Statistical significance was determined at a level of *P* < 0.05.

## Discussion

The metastatic spread of clear cell renal cell cancer (ccRCC) remains a formidable challenge, necessitating a comprehensive exploration of the intricate immune landscape [31, 32, 33]. In this study, we employed a powerful combination of single‐cell RNA sequencing (scRNA‐seq) analysis and immunofluorescent imaging to dissect the heterogeneity within CD8^+^ T cells, shedding light on their potential role in ccRCC metastasis.

Our scRNA‐seq analysis revealed a nuanced classification of CD8^+^ T cells into four distinct sub‐clusters, each characterized by unique genetic profiles. Gene set enrichment analysis provided crucial insights into the biological characteristics of these sub‐clusters, uncovering a subset of genes, including TYMP, TOP2A, CHI3L2, CDKN3, CENPM, and RZH2, that were markedly upregulated. Notably, the elevated expression of these genes within the identified sub‐clusters correlated strongly with a poor prognosis [34, 35, 36], indicating their potential as prognostic markers in ccRCC.

Among the identified sub‐clusters, CD8^+^ T cells characterized by the expression of MT‐ND4 emerged as a focal point of interest. The enriched expression of MT‐ND4 in these cells suggests a potentially pivotal role in mediating ccRCC metastasis. Understanding the molecular intricacies associated with MT‐ND4‐expressing CD8^+^ T cells could unveil novel therapeutic targets aimed at mitigating metastatic progression in ccRCC patients.

The findings presented in this study significantly contribute to our understanding of CD8^+^ T cell heterogeneity within the ccRCC microenvironment. The identification of distinct sub‐clusters and their associated genetic signatures not only refines our comprehension of the immune response against ccRCC but also opens avenues for the development of targeted therapeutic strategies. Moreover, the correlation between specific gene expression patterns and poor prognosis underscores the clinical relevance of our findings, emphasizing the potential utility of these genes as prognostic markers in ccRCC patients.

In conclusion, our integrative approach leveraging scRNA‐seq and immunofluorescent imaging has unraveled the complex landscape of CD8^+^ T cell heterogeneity in ccRCC [18, 37]. By elucidating the molecular underpinnings of these sub‐clusters, we provide valuable insights that may guide future therapeutic interventions, ultimately advancing our ability to combat ccRCC metastasis and enhance patient outcomes.

## Conflict of interest

The authors declare no conflict of interest.

### Peer review

The peer review history for this article is available at https://www.webofscience.com/api/gateway/wos/peer‐review/10.1002/2211‐5463.13819.

## Author contributions

LM and YL designed this study. YC, JS, and CL analyzed this scRNA‐seq data and helped compose the manuscript. ZL, SH, WL, and YQ helped perform this immunofluorescent imaging.

## Supporting information


**Table S1.** Genetic profile of CD8+ T cell sub‐populations.

**Table S2.** List of biocompare functional assay of CD8+ T cell sub‐clusters.

## Data Availability

The datasets and code developed or analyzed in this study were accessible upon sensible request from the corresponding author.
